# Supplementary material for: The semiquinone swing in the bifurcating electron transferring flavoprotein/butyryl-CoA dehydrogenase complex from Clostridium difficile
Source: Nat Commun. 2017 Nov 17;8:1577. doi: 10.1038/s41467-017-01746-3 (PMC5691135; doi:10.1038/s41467-017-01746-3)
Supplement: Supplementary file 1 — Supplementary Information [file 41467_2017_1746_MOESM1_ESM.pdf]

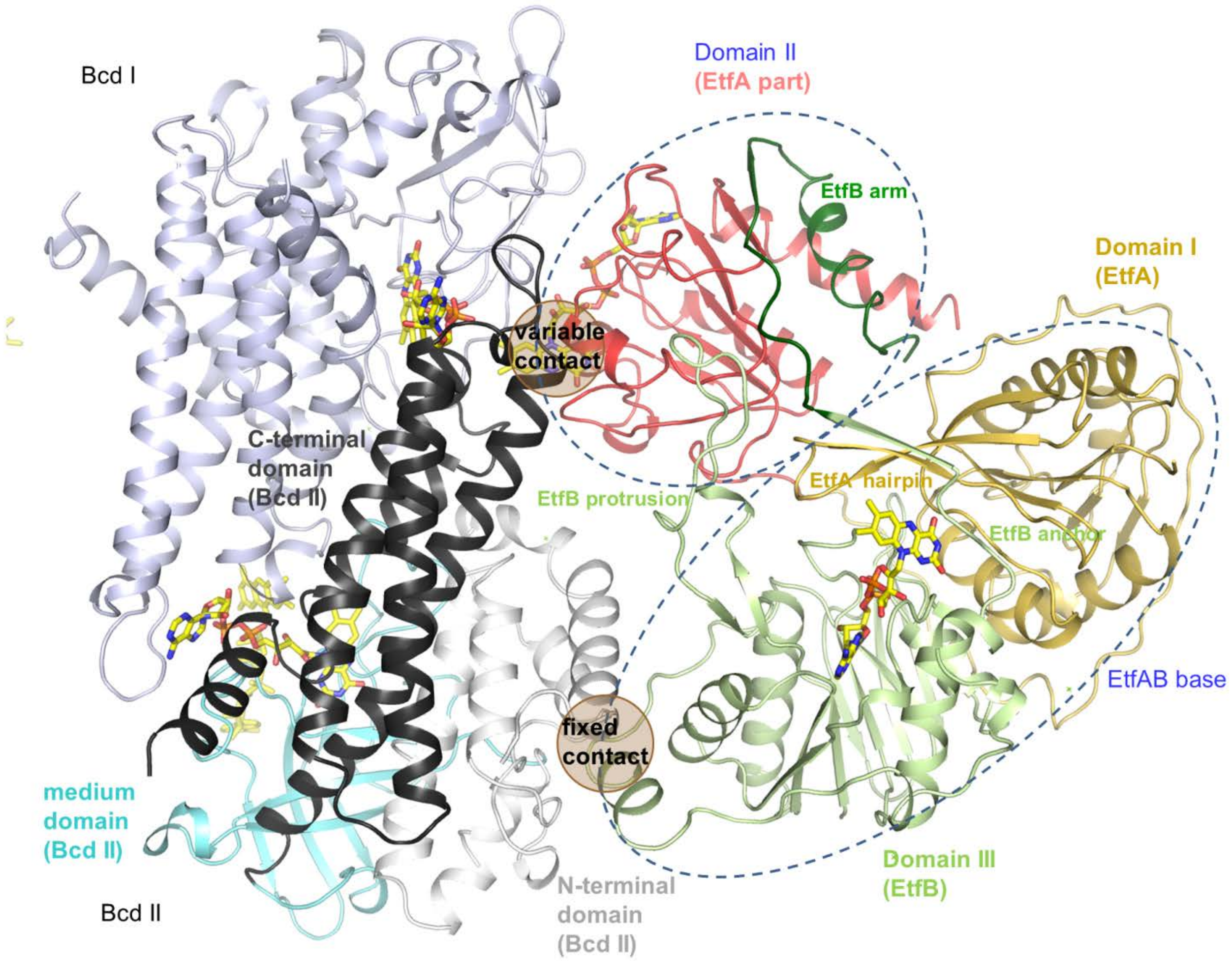

Supplementary Fig. 1 EtfAB/Bcd structure in the D state. One catalytic unit of the $(EtfAB/Bcd)_4$ complex consists of EtfAB, Bcd1 (light blue), and Bcd2 (N-terminal domain: light gray, medium domain in marine and C-terminal domain in dark gray). EtfAB consists of domain I (yellow) and domain III (light-green) forming the EtfAB base (marked by the larger blue circle), and domain II composed of the C-terminal part of EtfA (salmon) and the EtfB arm (dark green) (marked by the smaller blue circle).

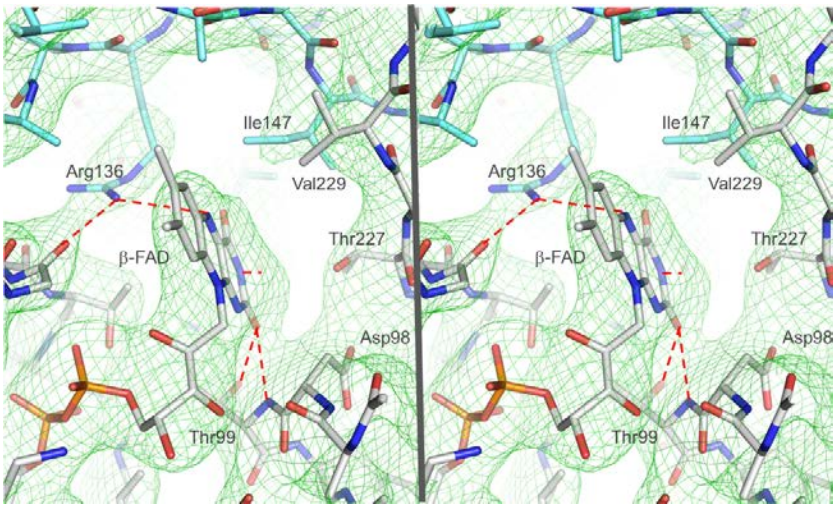

Supplementary Fig. 2 The electron bifurcating β-FAD and its surrounding. The $2F_o$-F_c electron density (green) is contoured at 1σ. The carbons of subunits α and β are drawn in lightcyan and gray.

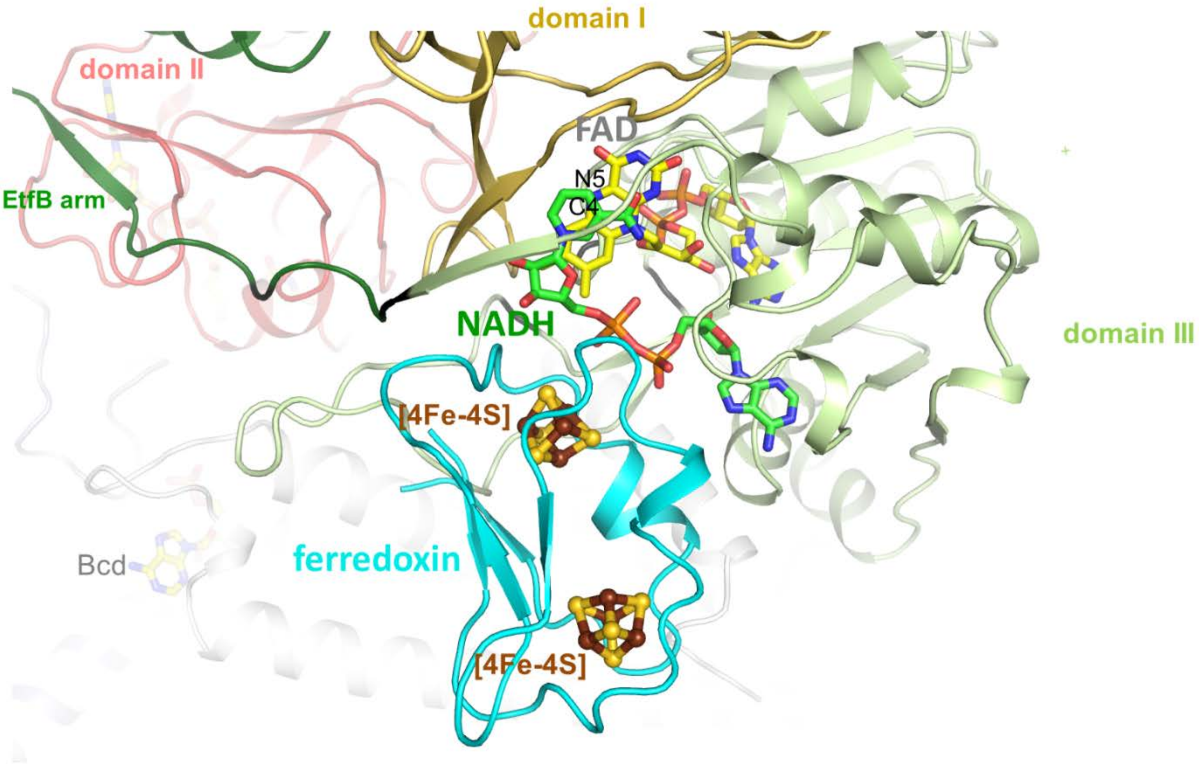

Supplementary Fig. 3 NADH and ferredoxin binding site. In the EtfAB-NADH complex structure of *A. fermentans* the ADP moiety was visible in the electron density. The ribose-nicotinamide moiety was modelled in a manner to position the N5 of FAD and C4 of NADH in van der Waals contact to each other. The ferredoxin was modeled to minimize the distance to β-FAD of EtfAB and the [4Fe-4S] cluster of ferredoxin without causing severe clashes between the polypeptide chains.

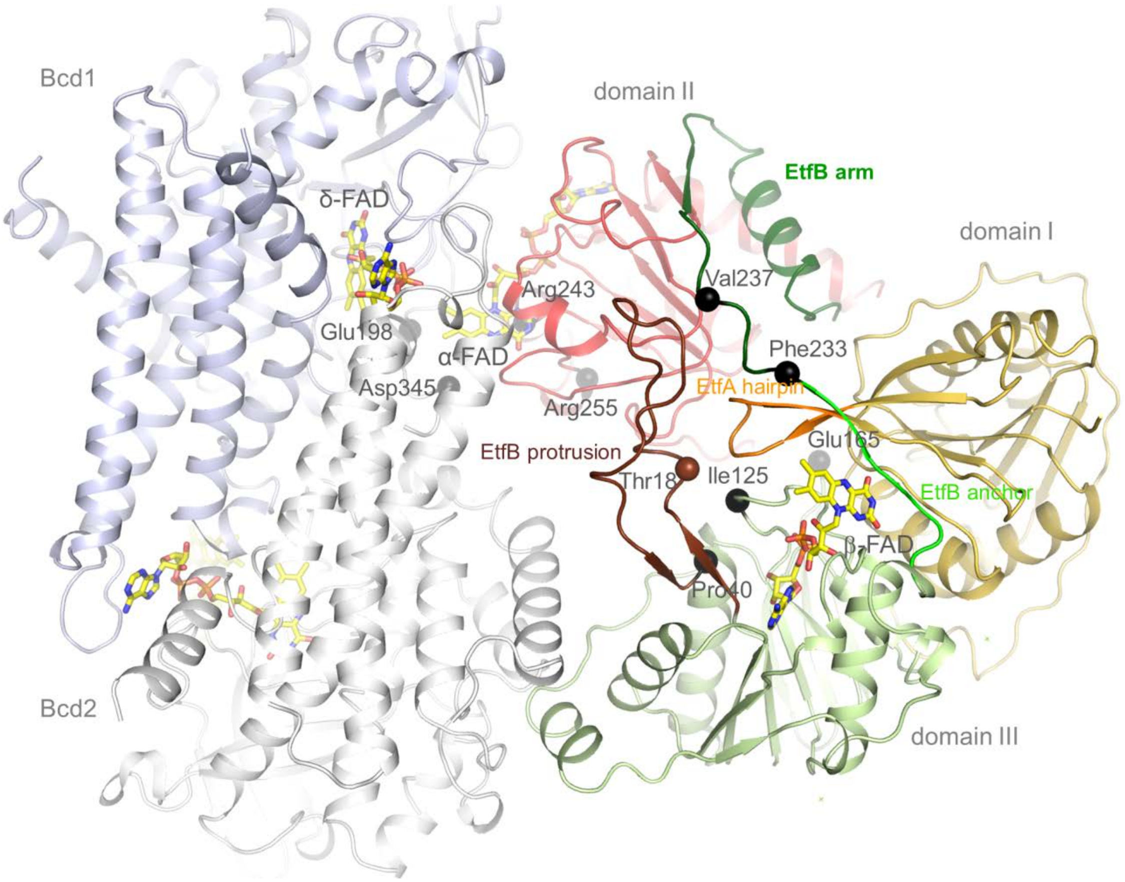

Supplementary Fig. 4 Location of the mutated residues in the EtfAB/Bcd complex. The Cα atoms of the mutated residues were marked as spheres.

Supplementary Table 1 Specific activities of the EtfAB/Bcd variants

**Assay**	**Bifurcation**	**Bcd /Fc^{3+}**	**NADH Oxidase**	**Remarks**
**Variant**	Specific activity (μmol × min^{-1} × mg^{-1} protein)			
**Wild type**	0.214	1.27	0.357	
Bcd E198Q	0.016	0.094	0.0259	conserved, interacts with EtfA R243 in the D-state
Bcd E198A	0.054	0.064	0.0189	
EtfA R243Q	0.011	0.027	0.0125	conserved, interacts with Bcd2 E198 in the D-state
Bcd D345N	0.0672	0.84	0.088	conserved, interacts with EtfA R243 in the D-state
Bcd D345A	0.130	0.95	0.190	
EtfB E165A	0.035	0.48	0.10	conserved, influences domain II rotation*
EtfB E165D	0.0396	0.315	0.041	
EtfA R255Q	**0.543**	**1.75**	**1.24**	not conserved, influences the EtfA linker
EtfA R255A	**0.249**	**1.36**	**0.629**	not conserved, influences the EtfA linker
EtfB T18E	0.0803	**3.13**	**0.899**	conserved in bifurcating Etfs, in protrusion
EtfB P40L	0.004	0.003	0.183	conserved in almost all Etfs, in protrusion
EtfB I125D	0.002	0.004	0.255	conserved, influences domain II rotation
EtfB I125F	0.007	0.24	0.0413	conserved, influences domain II rotation
EtfB F233A	**0.589**	**3.55**	**1.80**	conserved in bifurcating Etfs, H in *C. kluyveri,* in the anchor of the EtfB arm
EtfB V237N	0.0403	0.75	**0.72**	not conserved , in the EtfB linker

The kinetic characterization of the enzyme variants resulted in partly surprising results reflecting the complexity of the electron bifurcation process involving large-scale conformational changes which is influenced by many residues over a wide region. This might be the reason why almost each enzyme variant exhibits a strong modification of the kinetic parameters compared to that of the wild type. Even specific activities significantly higher (in boldface) than that of the wild type are found and rationalized by postulating an accelerated

rotation of domain II. Whereas the kinetic parameters of electron bifurcation can be plausibly interpreted, the influence of some mutations on the specific activity of NADH oxidation and butyryl-CoA reduction, which proceed in localized active sites remain elusive. The EtfB E165A mutant crystallized much faster than the wild type, probably due to inhibition of domain II rotation. Unfortunately, the crystals did not diffract properly.

Supplementary Table 2 Primers used for cloning of *etfAB-bcd* and the generation of mutants.

Cloning of *etfAB-bcd*

bcd forward: AAGCTCTTCAATGGATTTAAATTCTAAAAAATATCAGATGCT

etfA reverse: TGT GCTCTTCTCCCGTTAGCTAAAACTTCACCTTTTTCTTTTGC

Generation of mutants

	FORWARD	REVERSE
EtfαR136Q	ATTATTAATGACACAACCTG	CAAAGGCAGGTTGTGT
EtfαR136K	ATTATTAATGACAAAACCTG	CAAAGGCAGGTTTTGT
EtfαR136M	ATTATTAATGACAATGCCTG	CAAAGGCAGGCATTGT
EtfαR243Q	GTTTCTGGTTCTCAAGCCACTATA	CCTGCATCTATAGTGGCTTGAGAAC
ETfαR255Q	TTAGATAAAGCACAACAAGTT	GTTTGACCAACTTGTTGTGCTTTA
EtfβT18E	CAAGTTCCAGATACAGAAGAAG	GGATCTAGTTTAACTTCTTCTGTAT
EtfβP40L	CAAGTATAATAAACCTTGATG	TGCTTTATCATCAAGGTTT
EtfβI124D	GAAGACAGGCGGATGATG	CAGTATCTCCATCATCCGC
EtfβI124F	GAAGACAGGCGTTTGATG	CAGTATCTCCATCAAACGC
Etfβ E165A	GTAAAAAGACAATTTGCAGATTGT	TAAGTCATGGCAACAATCTGCAAATTG
EtfβE165D	GTAAAAAGACAATTTGACGATTGT	TCATGGCAACAATCGTCAAATTG
EtfβF232A	GTGTATTTAAATCAGCTACAA	TTAACTGATTTTGTAGCTGAT
EtfβV236N	CATTTACAAAATCAAATAAACC	CCAGCTGGTTTATTTGATT
BcdE198Q	TTGGAGTTAAACAAAAG	CCCATTTTCTTTTGTTTA
BcdE198A	TTGGAGTTAAAGCAAAG	CCCATTTTCTTTGCTTTA
BcdD345N	GATACACTCGTAACTATC	TTCTACTGGATAGTTACGAG
BcdD345A	GATACACTCGTGCCTATC	TTCTACTGGATAGGCACGAG
